# Supplementary material for: Nonperturbative semiclassical spin dynamics for ordered quantum magnets
Source: NPJ Quantum Mater. 2026 May 16;11(1):75. doi: 10.1038/s41535-026-00873-9 (PMC13518224; doi:10.1038/s41535-026-00873-9)
Supplement: Supplementary file 1 [file 41535_2026_873_MOESM1_ESM.pdf]

Supplementary Material For "Nonperturbative Semiclassical Spin Dynamics for Ordered Quantum Magnets"

Hao Zhang,[1] Tianyue Huang,[2] Allen O. Scheie,[3] Mengze Zhu,[4] Tao Xie,[5] N. Murai,[6] S. Ohira-Kawamura,[6] Andrey Zheludev,[4] Andreas M. Läuchli,[7,2] and Cristian D. Batista[1,8]

[1]*Department of Physics and Astronomy, The University of Tennessee, Knoxville, Tennessee 37996, USA*
[2]*Institute of Physics, École Polytechnique Fédérale de Lausanne (EPFL), CH-1015 Lausanne, Switzerland*
[3]*MPA-Q, Los Alamos National Laboratory, Los Alamos, New Mexico 87545, USA*
[4]*Laboratory for Solid State Physics, ETH Zürich, 8093 Zürich, Switzerland*
[5]*Center for Neutron Science and Technology, Guangdong Provincial Key Laboratory of Magnetoelectric Physics and Devices, School of Physics, Sun Yat-sen University, Guangzhou, Guangdong 510275, China*
[6]*J-PARC Center, Japan Atomic Energy Agency, Tokai, Ibaraki 319-1195, Japan*
[7]*Laboratory for Theoretical and Computational Physics, Paul Scherrer Institut, CH-5232 Villigen-PSI, Switzerland*
[8]*Quantum Condensed Matter Division and Shull-Wollan Center, Oak Ridge National Laboratory, Oak Ridge, Tennessee 37831, USA*

(Dated: February 20, 2026)

I. CONSTANT-$\boldsymbol{q}$ CUTS OF INTENSITY DATA

In this section, we present constant-$\boldsymbol{q}$ cuts of the intensity data obtained from inelastic neutron scattering (INS) experiments, as well as from truncated Hilbert space exact diagonalization (THED) and matrix product state (MPS) calculations, as shown in Figs. 1, 4, and 5 of the main text. These detailed comparisons allow for a clearer visualization of the agreement among the data and facilitate a more precise analysis of their discrepancies as discussed in Sec. II.

A. Materials: $KYbSe_2$, $CsYbSe_2$, and $K_2Co(SeO_3)_2$

To enable a quantitative comparison with the INS data of $KYbSe_2$, we apply both the neutron polarization factor and the $\boldsymbol{g}$-tensor specific to $KYbSe_2$ to the dynamical spin structure factor (DSSF) computed using THED [Eq. 9 in Methods] and MPS [Eq. 13 in Methods]. The resulting INS intensity is given by

$$I(\boldsymbol{q},\omega) = \sum_{\mu=x,y,z} \left(1 - \frac{q^\mu q^\mu}{|\boldsymbol{q}|^2}\right) (g^{\mu\mu})^2 \mathcal{S}^{\mu\mu}(\boldsymbol{q},\omega), \tag{S1}$$

where $g^{xx} = g^{yy} = 3.02$ and $g^{zz} = 1.86$ are the components of the $\boldsymbol{g}$-tensor reported in Ref. [1], and $\mathcal{S}^{\mu\mu}(\boldsymbol{q},\omega)$ denotes the diagonal components of the DSSF. Due to experimental challenges, the determination of the g-factors carries significant uncertainties [1]. In our THED and MPS calculations, we fix the Zeeman energy as $g^{xx}\mu_B B = 1.73J_1$ ($J_1 = 0.456$ meV and α is the field direction) to simulate the INS data at $B = 4$ T, and subsequently apply the g-tensor corrections to the calculated DSSF. We emphasize that only these diagonal terms contribute to the INS intensity for the present system.

The resulting comparison among INS, THED, and MPS data is presented as heatmaps in Fig. 1**d** of the main text. Here we focus on three representative $\boldsymbol{q}$ points along the $[q_1, q_1, 3]$ direction, specifically at $q_1 = 0.15$, $1/3$, and 0.5. To highlight the role of magnon-magnon interactions, we also include results from the non-interacting two-magnon continuum, computed using Eq. 4 in the Methods with the interaction vertex $\tilde{U}$ in Eq. 7 set to zero.

As shown in the first two rows of Fig. S1, both THED and MPS closely reproduce the INS data for $KYbSe_2$, capturing the peak positions and widths with high fidelity. To disentangle the different contributions to the DSSF, we show the transverse channel, which probes single-magnon excitations, in the third row, and the longitudinal channel, which captures two-magnon contributions in the fourth row. For consistent quantitative comparison, the non-interacting, THED, and MPS data in Fig. S1 and Fig. 1**d** of the main text have been convolved with a Gaussian resolution function of width $\sigma = 0.1J_1^{\text{kys}}$.

Minor discrepancies between THED and MPS arise in the single-magnon peaks at $q_1 = 0.15$ and 0.5, likely due to three-magnon contributions not included in the THED framework. Notably, at $q_1 = 1/3$, where the single-magnon mode is well separated from the three-magnon continuum (see Sec. II for further discussion), the agreement between the two methods is excellent.

The longitudinal spectra in the fourth row of Fig. S1 exhibit similar consistency between THED and MPS, with only minor deviations arising from the same truncation limitations in THED. Importantly, both methods reveal marked deviations from the non-interacting two-magnon continuum, highlighting the substantial role of magnon-magnon interactions in reshaping the spectral lineshape. This provides clear evidence of pronounced non-perturbative effects in $KYbSe_2$.

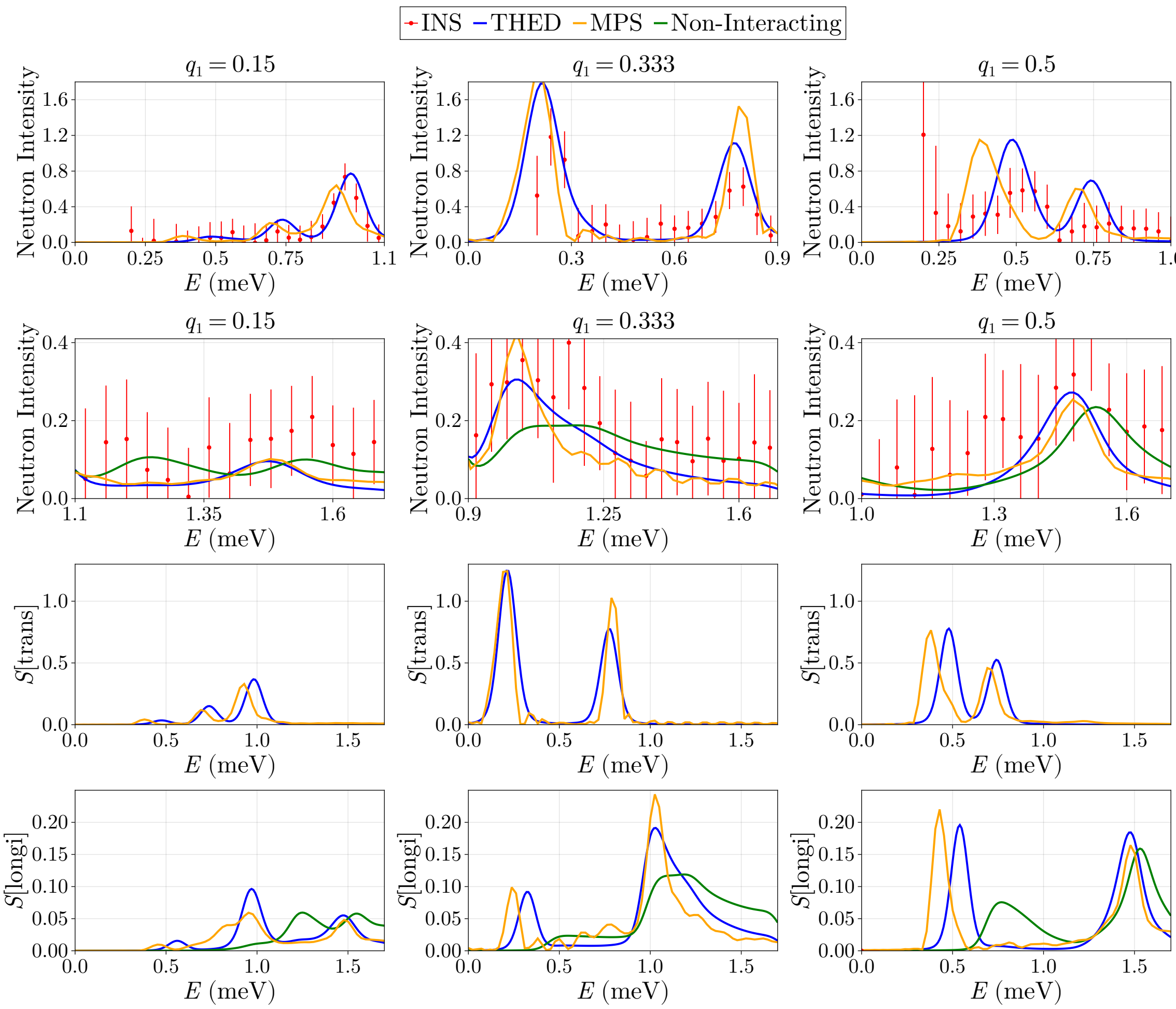

FIG. S1. Comparison of constant-$\boldsymbol{q}$ cuts for $KYbSe_2$ at three representative momenta along the $[q_1, q_1, 3]$ direction. **Rows 1–2:** Inelastic neutron scattering (INS) intensity versus theoretical results. Row 1 highlights the low-energy sector dominated by single-magnon contributions, compared with truncated Hilbert space exact diagonalization (THED) and matrix product state (MPS) calculations. Row 2 focuses on the high-energy sector dominated by two-magnon contributions, compared with THED, MPS, and the non-interacting two-magnon continuum. **Row 3:** Transverse dynamical spin structure factors from THED and MPS. **Row 4:** Longitudinal dynamical spin structure factors from THED, MPS, and the non-interacting two-magnon continuum.

We note that the two-magnon bound state appears as a weak, low-lying feature–visible as the first peak in all three panels of the bottom row–located in close proximity to the single-magnon peak. Its low intensity and overlap with nearby excitations render it challenging to resolve unambiguously in the INS data.

Figure S2 presents constant-$\boldsymbol{q}$ cuts of the intensity data for both $CsYbSe_2$ and $K_2Co(SeO_3)_2$. To enable quantitative comparison, the simulated data shown here and in Fig. 5 of the main text have been convoluted with a Gaussian wave packet of width $\sigma = 0.1J_1$, where J_1 denotes the nearest-neighbor interaction for the corresponding compound. Due to the strong anisotropy in the g-tensor of these materials–$g_{ab} = 3.25$ and $g_{cc} = 0.2$ for $CsYbSe_2$ (with in-plane UUD order), and $g_{ab} = 2$, $g_{cc} = 7.9$ for $K_2Co(SeO_3)_2$ (with UUD order along the c-axis)–the longitudinal component of the DSSF can be effectively extracted from the INS data. As discussed in the main text, for materials with a weak easy-plane exchange anisotropy, we neglect this small contribution and model the system using a pure Heisenberg interaction. This approximation preserves the U(1) symmetry of $\mathcal{H}$ for arbitrary field orientations.

In the top row of Fig. S2, both THED and MPS show good quantitative agreement with the INS spectra for $CsYbSe_2$. In the middle row, we display only the simulated longitudinal DSSF, as the corresponding component extracted from the INS data is too noisy to allow a meaningful comparison. A clearer illustration of the agreement between simulation and experiment can be found in the heatmap shown in Fig. 5 of the main text.

Finally, in the bottom row of Fig. S2, THED and MPS results exhibit excellent agreement for $K_2Co(SeO_3)_2$, which lies deep in the Ising regime with $\Delta = J_{zz}/J_{xy} = 14.28$, further validating the accuracy and consistency of both theoretical approaches in

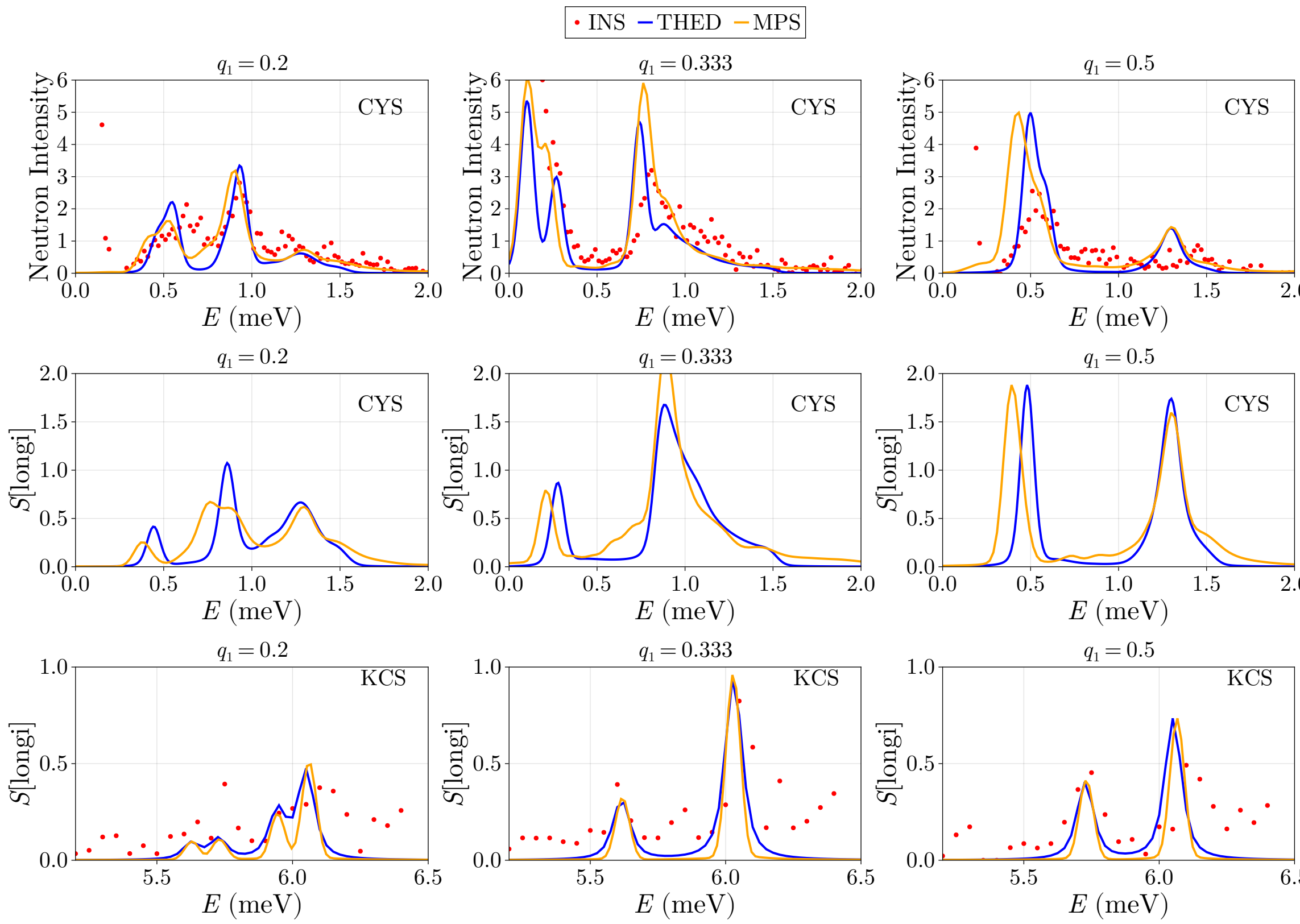

FIG. S2. Comparison of constant-$\boldsymbol{q}$ cuts for $CsYbSe_2$ and $K_2Co(SeO_3)_2$. Top row: INS data compared with simulated results from THED and MPS along the momentum path $[q_1, q_1, 2.5]$. Middle row: Longitudinal component of the dynamical spin structure factor for $CsYbSe_2$. Bottom row: Longitudinal component of the dynamical spin structure factor for $K_2Co(SeO_3)_2$.

this limit.

B. XXZ Hamiltonian

Figure S3 presents a comparison of constant-$\boldsymbol{q}$ cuts of the longitudinal DSSF for the XXZ model at three representative anisotropy values: $\Delta = 5$, 2, and 1. As with the other data sets, both the THED and MPS results are convolved with a Gaussian resolution function of width $\sigma = 0.1J$. These results correspond to those shown in Fig. 4 of the main text. The MPS data were obtained by time-evolving a cylindrical system of size $L_x = 27, L_y = 8$ with bond dimension $\chi = 1024$. The rationale for using a wider cylinder for the XXZ Hamiltonian is provided in Sec. II. Across the entire range, from the Ising limit to the Heisenberg point, we find good agreement between the two methods, with only minor discrepancies, which are discussed in detail below.

II. ANALYSIS OF DISCREPANCIES

In this section, we analyze the potential sources of discrepancies between the THED and MPS results, which may arise from limitations inherent to the current implementations of both methods. In particular, the truncation of $n_{\text{mag}} \geq 3$ magnon states in THED and the use of cylindrical geometries in MPS calculations represent the primary sources of deviation.

A. Truncation of multi-magnon states

To estimate the contributions of $n_{\text{mag}} \geq 3$ states, Fig. S4 displays the one-, three-, two-, and four-magnon states in four columns, with different rows corresponding to exchange anisotropies $\Delta = 5, 2, 1$. The one- and two-magnon states are obtained directly

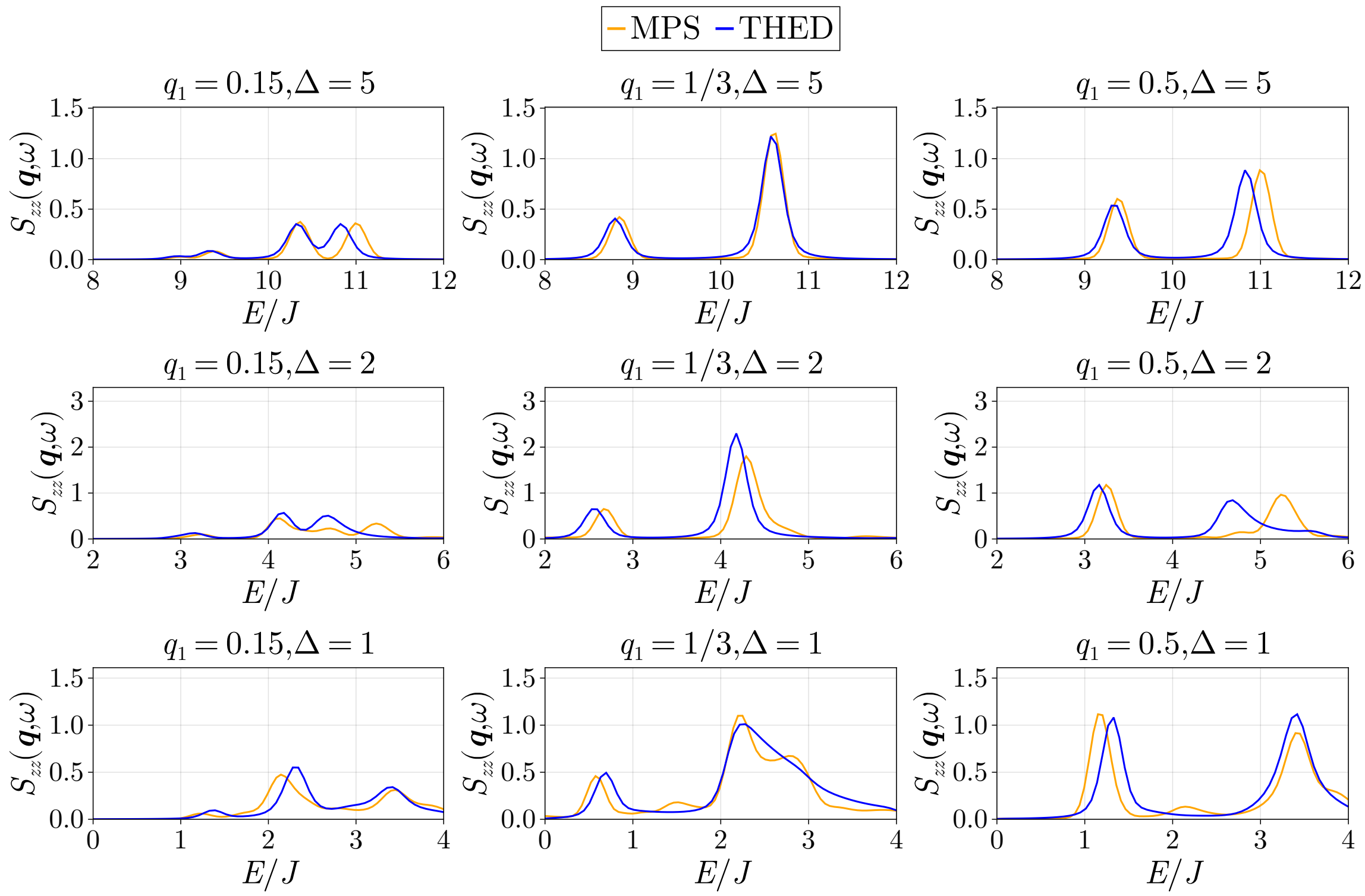

FIG. S3. Comparison of constant-$\boldsymbol{q}$ cuts of the longitudinal dynamical spin structure factor for the XXZ model at different anisotropy parameters Δ. THED results are shown alongside MPS data.

from THED up to two-magnon states, whereas the three- and four-magnon continua are constructed from the one-magnon states and the lowest two-magnon bound state band, and from the two two-magnon bound states from the lowest band, respectively.

If the Hilbert space is extended to include contributions up to four magnons, then, in addition to the particle-number conserving term $\beta^\dagger\beta^\dagger\beta\beta$ already incorporated in the current THED implementation, one must also account for particle-number non-conserving quartic interaction vertices of the form $U^{(4)}_{\text{p-non}} \sim \beta^\dagger\beta\beta\beta$. These vertices connect one- to three-magnon states and two- to four-magnon states, provided the sectors share the same quantum number ΔS^z (color-coded in Fig. S4). Since the model under consideration lacks a cubic vertex, no matrix elements exist between sectors with different magnon-number parity (e.g. between one- and two-magnon states or between one- and four-magnon states).

The small but systematic downshift of the single-magnon peak positions at the Heisenberg point $\Delta = 1$ can be attributed to $U^{(4)}_{\text{p-non}}$ through level repulsion. As shown in the third row of Fig. S1 and the first row of Fig. S2, the downshift is smaller for $q_1 = 1/3$ compared to $q_1 = 0.15$ and $q_1 = 0.5$, consistent with the fact that $q_1 = 1/3$ lies further from the three-magnon continuum (see Fig. S4).

Moreover, as shown in Fig. S3, interactions between two- and four-magnon states mediated by $U^{(4)}_{\text{p-non}}$ may also contribute to the discrepancies between the longitudinal DSSF obtained from THED and MPS. Specifically, the MPS calculation reveals a weak intensity mode in the energy window $1.5 \lesssim E/J \lesssim 2$ at $\Delta = 1$ along the $[q_1, q_1, 0]$ direction for $q_1 \simeq 0.2 - 0.5$, which is absent in the THED results. As indicated in Fig. S4, this mode coincides with the lower edge of the estimated four-magnon continuum formed by two two-magnon bound states. This suggests that the additional feature in MPS may correspond either to a four-magnon bound state or to the onset of the two two-magnon bound state continuum. Such an interpretation would also explain its absence in the current THED framework, which includes only single- and two-magnon states. However, as shown in Fig. S5, this mode is not observed in MPS calculations for $L_y = 6$, while a similar feature does emerge in THED calculations for cylindrical geometries with small aspect ratios (see Fig. S6). Consequently, the discrepancy could also be attributed to finite size effects.

At $\Delta = 2$, discrepancies in the longitudinal DSSF arise in the energy range $4.5 \lesssim E/J \lesssim 5.5$. In this window, the MPS results exhibit a splitting between two modes for $H < 1/3$, whereas THED shows only a single branch. For $H > 1/3$, both approaches yield a dominant high-intensity mode, but the MPS mode lies at systematically higher energy. Again, as shown in Fig. S4, this interval coincides with the overlap of two-magnon states and the four-magnon continuum, highlighting the role of higher-magnon sectors in accounting for these differences.

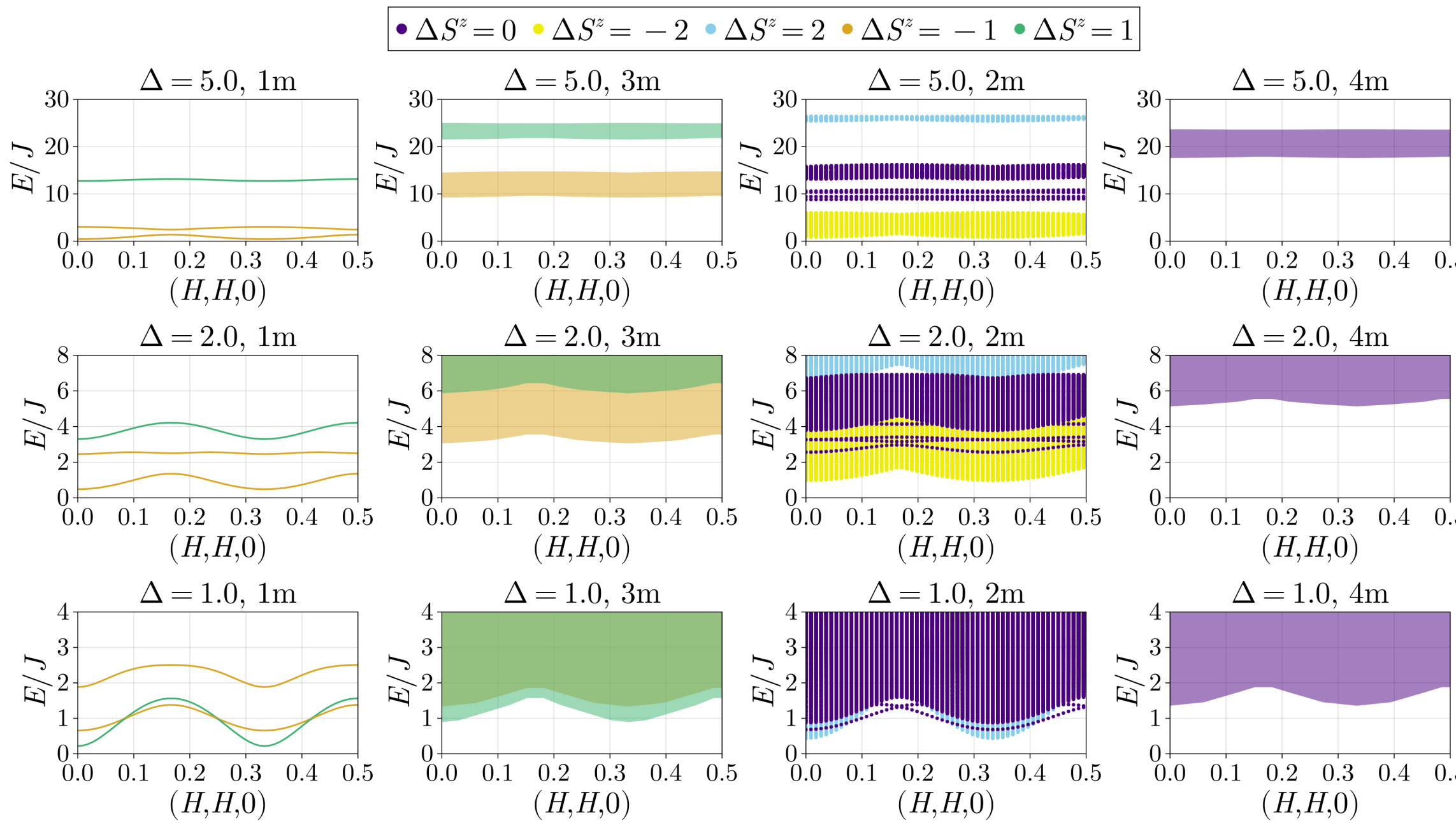

FIG. S4. One- to four-magnon states estimated from THED. The first two columns show the one- and three-magnon states, while the last two columns show the two- and four-magnon states. Different rows correspond to exchange anisotropies $\Delta = 5, 2, 1$. The one- and two-magnon states are obtained directly from THED up to two-magnon states, whereas the three- and four-magnon continua are constructed from the one-magnon states and the lowest two-magnon bound state band, and from the two two-magnon bound states from the lowest band, respectively. The color indicates the quantum number ΔS^z of the corresponding state.

The overall good agreement between THED and MPS indicates that the contribution of $U^{(4)}_{\text{p-non}}$ is minor for this problem. As discussed below, the discrepancies in the longitudinal DSSF shown in Fig. S3 may also arise from the cylindrical geometry employed in MPS. Extending the current THED implementation to include the four-magnon sector increases the computational cost only from linear to cubic in system size, making the incorporation of three- and four-magnon states a promising and tractable avenue for future work to further reduce truncation errors and clarify the origin of these discrepancies.

B. The impact of cylindrical geometry

Our MPS calculations are carried out on cylindrical geometries, with open boundaries along the x direction of length L_x and periodic boundaries along the y circumference of length L_y. A key assumption is that the entanglement growth along the effective one-dimensional mapping remains manageable, which typically requires a moderate circumference. To examine the impact of geometry, we computed the longitudinal DSSF of the XXZ model for cylinders with $L_y = 6$ and $L_y = 8$. The convergence with respect to bond dimension is shown in Fig. 7 for $L_y = 6$ ($\chi = 512$) and Fig. 8 (in the Methods) for $L_y = 8$ ($\chi = 1024$). A direct comparison of the longitudinal DSSF for the two cylinder widths is given in Fig. S5, which reveals that even this modest increase in circumference leads to noticeable shifts in peak positions and splittings at certain wave vectors.

To further assess finite-size effects, we performed additional THED calculations on clusters with varying aspect ratios, all with periodic boundary conditions in both directions. We find that the longitudinal DSSF S^{zz} is highly sensitive to the aspect ratio, particularly at $\Delta = 2$ and $\Delta = 1$. As shown in Fig. S6, the cylindrical geometry can induce spectral-weight splittings in these cases, coinciding with the largest discrepancies between the two methods–for example, at $\Delta = 2$, $q_1 = 0.5$, and $\Delta = 1$, $q_1 = 1/3$. Calculations with smaller aspect ratios exhibit spurious splittings compared to results closer to the thermodynamic limit.

In summary, while some of the discrepancies between THED and MPS can be attributed to the truncation of $n_{\text{mag}} \geq 3$ states in THED, they may also originate from the cylindrical geometry used in MPS. These findings highlight the need for methodological improvements in both approaches to resolve such ambiguities. Overall, the detailed comparison presented here reinforces that the combined use of THED and MPS provides a powerful framework for understanding non-perturbative spin dynamics in ordered quantum magnets.

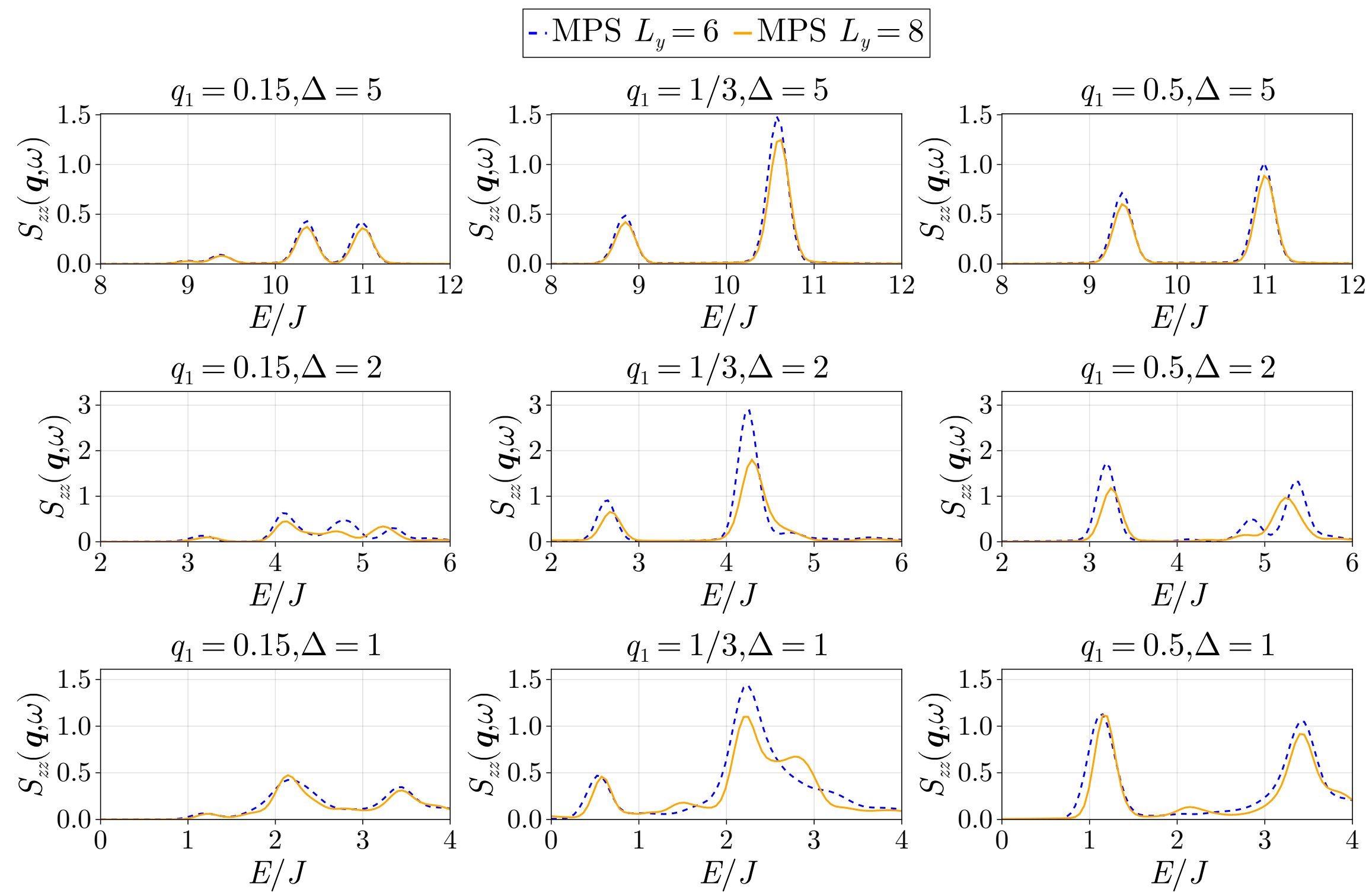

FIG. S5. Longitudinal dynamical spin structure factor at constant-$\boldsymbol{q}$ from MPS simulations with $L_y = 6$ and $L_y = 8$ for three exchange anisotropies.

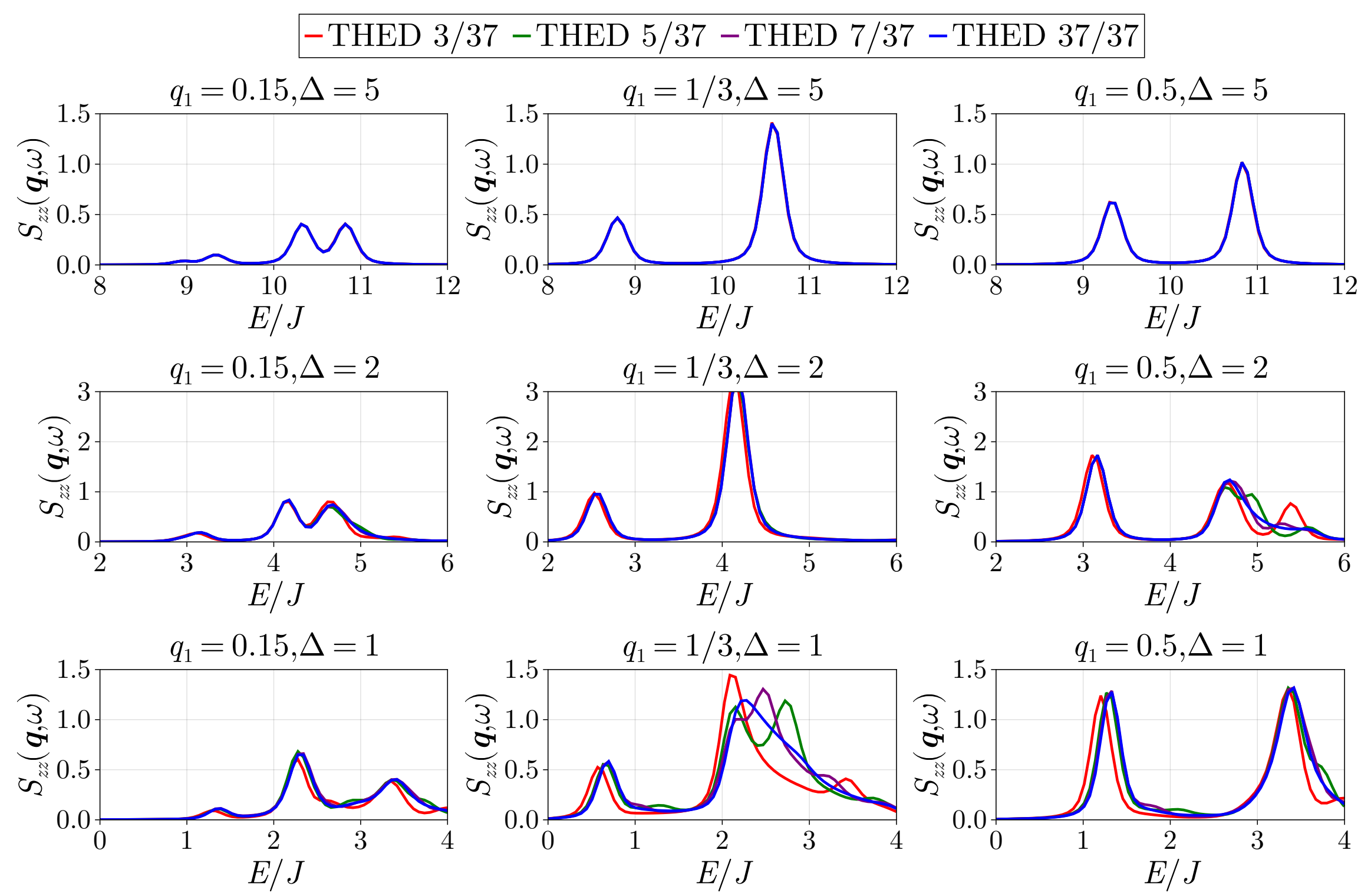

FIG. S6. THED calculations in a cylinder geometry with different aspect ratios of magnetic unit cells along two directions with periodic boundary conditions.

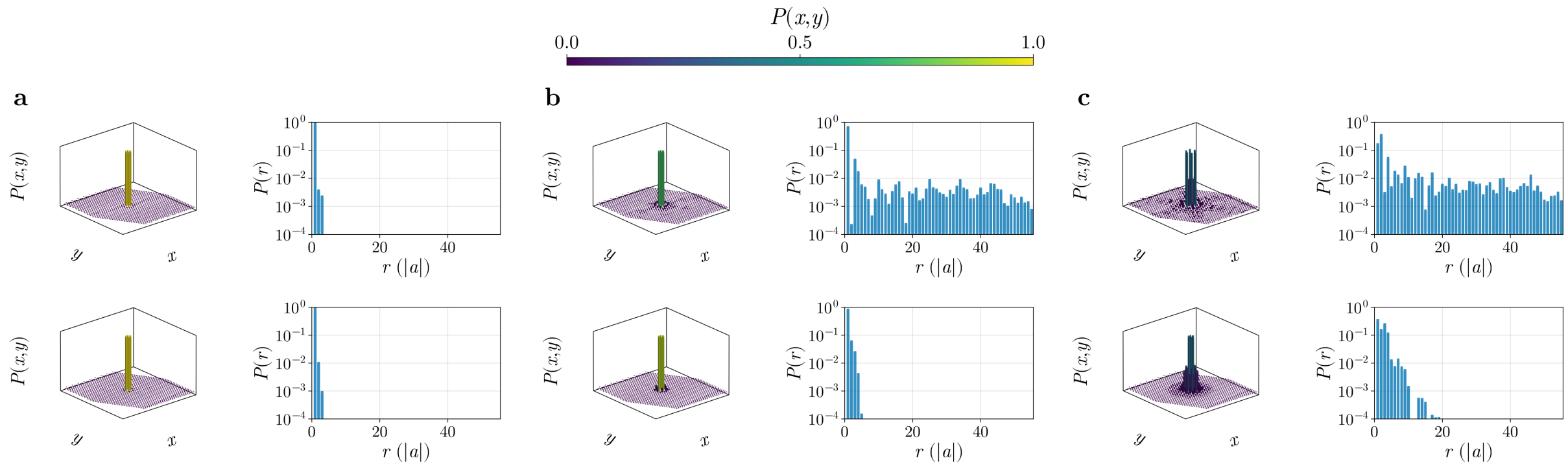

FIG. S7. Squared real-space wave functions for the two states with the highest spectral intensity at center-of-mass momentum $\boldsymbol{q} = [1/3, 1/3, 0]$ shown in Fig. 4 of the main text. The top row corresponds to the state with the highest intensity on the upper branch, while the bottom row shows results for the state on the lower branch. **a** ($\Delta = 5$), **b** ($\Delta = 2$), **c** ($\Delta = 1$): The first column displays the spatial distributions of the squared wave functions in linear coordinates. The second column shows angle-integrated results on a semi-logarithmic scale to highlight the asymptotic behavior.

III. INTERPRETABILITY AND REAL SPACE WAVE FUNCTION

We now demonstrate that the THED method offers valuable interpretability through direct access to the eigenstates of the truncated Hamiltonian. Let us begin with the unnormalized two-spin-flip wave function

$$\psi(\mathbf{0}, \boldsymbol{r}; \alpha, \beta) = b^{\dagger}_{\mathbf{0}\alpha} b^{\dagger}_{\boldsymbol{r}\beta} |0\rangle, \tag{S2}$$

which represents the probability amplitude for creating one spin flip at the origin (magnetic unit cell $\mathbf{0}$) on sublattice α and another at site $\boldsymbol{r}$ on sublattice β. Here, $b^{\dagger}_{\alpha}$ creates the Holstein-Primakoff boson in the local frame of the αth sublattice, and $|0\rangle$ is the vacuum of the Bogoliubov quasiparticles $\beta^{\dagger}$ (see Eq. 4 of Methods).

The eigenstates from exact diagonalization of the truncated Hamiltonian are labeled by center-of-mass momentum $\boldsymbol{q}$ and band index $\tilde{n}$, i.e., $|\boldsymbol{q}, \tilde{n}\rangle$. We compute the squared overlap between this eigenstate and the real-space wave function:

$$|\langle \boldsymbol{q}, \tilde{n} | \psi(\mathbf{0}, \boldsymbol{r}; \alpha, \beta)\rangle|^2 = \left| \frac{1}{N_u} \sum_{\boldsymbol{k}\prime} \frac{U^*_{\tilde{n}}(\boldsymbol{q}, \boldsymbol{k})}{\zeta(\boldsymbol{q}-\boldsymbol{k}, n_1; \boldsymbol{k}, n_2)} \left[e^{-i(\boldsymbol{q}-\boldsymbol{k})\cdot\boldsymbol{r}} [V_{\boldsymbol{k}}]^*_{\alpha, n_2} [V_{\boldsymbol{q}-\boldsymbol{k}}]^*_{\beta, n_1} + e^{-i\boldsymbol{k}\cdot\boldsymbol{r}} [V_{\boldsymbol{q}-\boldsymbol{k}}]^*_{\alpha, n_1} [V_{\boldsymbol{k}}]^*_{\beta, n_2} \right] \right|^2, \tag{S3}$$

where $U_{\tilde{n}}(\boldsymbol{q}, \boldsymbol{k})$ is the eigenvector of the truncated Hamiltonian corresponding to band $\tilde{n}$, and $[V_{\boldsymbol{k}}]_{\alpha, n_1}$ denotes the Bogoliubov transformation matrix element that para-diagonalizes Eq. 4 of Methods, with α, β labeling the sublattice and n_1, n_2 the single-magnon band index. The prime on the momentum summation indicates that it is restricted to two-particle states with fixed center-of-mass momentum $\boldsymbol{q}$.

In Fig. S7, we plot the (normalized) squared wave functions obtained from Eq. (S3) for the two states with the highest spectral weight at $\boldsymbol{q} = (1/3, 1/3, 0)$, as shown in Fig. 4 of the main text. For bound states, the probability of finding the second spin flip decays exponentially beyond a characteristic binding length. In contrast, for resonant states, this probability remains finite even at large separations, giving rise to long tails in the distribution.

This distinction is made clearer in the angle-integrated plots shown on a semi-logarithmic scale. The bound states exhibit clear exponential decay within the finite cluster, while the resonance states display extended profiles, indicating delocalization. These observations reinforce the physical picture that THED captures both bound and resonance states, offering a powerful framework for interpreting features in the dynamical spin structure factor.

Direct access to the real-space wavefunction allows us to explore an instructive scenario: we begin by creating a spin flip at the origin, $\boldsymbol{r}_{10} = \mathbf{0}$, on sublattice α_0, followed by a second spin flip at a neighboring site, $\boldsymbol{r}_{20} = \boldsymbol{r}_{10} + \boldsymbol{r}_0$ (with $\boldsymbol{r}_0 = \boldsymbol{a}$), on sublattice β_0. This initial configuration is not an eigenstate of the truncated Hamiltonian and thus evolves under its dynamics.

To probe this evolution, we evaluate the probability of finding the second spin flip at a relative displacement $\boldsymbol{r} = \boldsymbol{r}_2 - \boldsymbol{r}_1$ with respect to the first, at a given time t. This is computed through the transition amplitude at fixed center-of-mass momentum $\boldsymbol{q} = (1/3, 1/3, 0)$:

$$\begin{aligned} C(\boldsymbol{r}, t; \boldsymbol{r}_0, \alpha, \beta, \alpha_0, \beta_0) &= \langle \psi(\boldsymbol{r}; \alpha, \beta) | e^{-i\tilde{H}_{\boldsymbol{q}} t} | \psi(\boldsymbol{r}_0; \alpha_0, \beta_0)\rangle \\ &= \sum_{\tilde{n}} e^{-i\mathcal{E}_{\boldsymbol{q}, \tilde{n}} t} \langle \psi(\boldsymbol{r}; \alpha, \beta) | \boldsymbol{q}, \tilde{n}\rangle \langle \boldsymbol{q}, \tilde{n} | \psi(\boldsymbol{r}_0; \alpha_0, \beta_0)\rangle \end{aligned} \tag{S4}$$

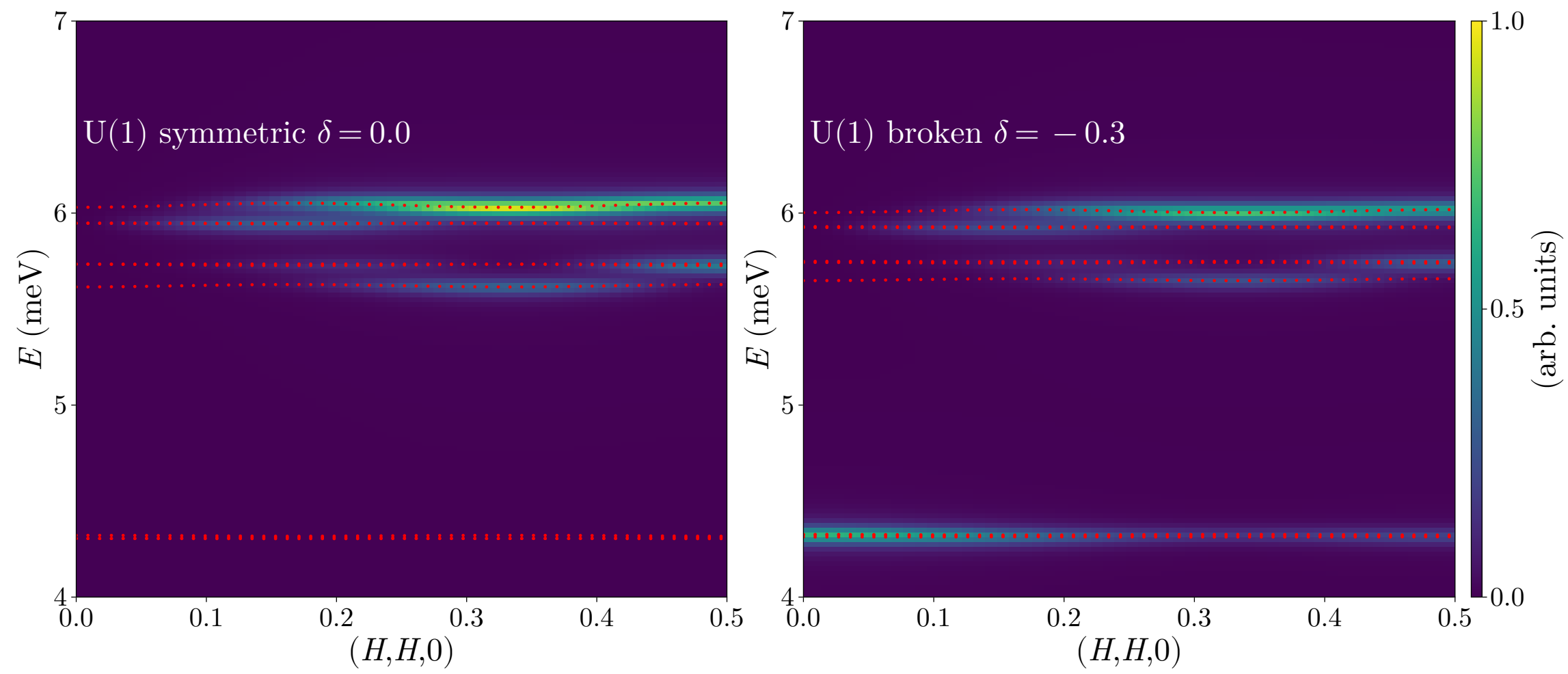

FIG. S8. Effect of U(1) symmetry breaking on the INS spectrum of $K_2Co(SeO_3)_2$. The red dots indicate the eigenstates of the truncated Hamiltonian, overlaid on the INS intensity calculated using the THED method. The left panel shows results for the U(1)-symmetric case ($\delta = 0$), while the right panel shows the spectrum after introducing a finite U(1)-breaking anisotropy ($\delta \neq 0$). Upon breaking U(1) symmetry, the quadrupolar bound state–previously invisible–gains dipolar character and becomes detectable via INS.

where $\tilde{H}_{\boldsymbol{q}}$ denotes the truncated Hamiltonian introduced in Eq. 5 of Methods, evaluated at $\boldsymbol{q} = (1/3, 1/3, 0)$, and $\mathcal{E}_{\boldsymbol{q},\tilde{n}}$ labels the eigenvalues of $\tilde{H}_{\boldsymbol{q}}$.

In the videos submitted with this article "*dynamical_wave_function_Δ_(5.0,1.0).mp4*," we visualize the time evolution of the quantum state $\psi(\boldsymbol{r}_0; \alpha_0, \beta_0)$ by plotting the square of the absolute value of the transition amplitude defined in Eq. (S4) (after normalization). In the UUD phase, the Ising limit ($\Delta = 5$) clearly exhibits two spin flips that remain tightly bound over time, with occasional quantum tunneling of the second spin flip to a different nearest-neighbor site.

In contrast, at the Heisenberg point ($\Delta = 1$), the two spin flips form a transient bound pair that persists over a finite lifetime $\tau \sim \hbar/|\Gamma|$, where Γ is the average width (over momentum) of the resonance in $\mathcal{S}^{\mu\mu}(\boldsymbol{q}, \omega)$. Beyond this time scale, the pair decays into two independent magnons.

We anticipate that in situ correlation measurements, such as those proposed in Ref. [2], could directly detect this real-space, time-resolved evolution of the quantum states, as illustrated in the simulations and accompanying video files.

IV. U(1) SYMMETRY BREAKING TERM

As discussed in the main text, the two-magnon bound states shown in Fig. 3**d** are invisible to INS experiments due to their quadrupolar nature. This conclusion, however, relies on the assumption that the model Hamiltonian preserves the U(1) symmetry of global spin rotations along the magnetic field axis, as is the case for Eq. 1 in the main text. We now explicitly break the U(1) symmetry by introducing an additional anisotropy (allowed by symmetry) in the $S^y S^y$ term:

$$\mathcal{H} = J \sum_{\langle i,j \rangle} \left(S_i^x S_j^x + (1+\delta) S_i^y S_j^y + \Delta S_i^z S_j^z \right) - g_{cc} \mu_B \sum_i S_i^z . \tag{S5}$$

The parameter δ quantifies the strength of U(1) symmetry breaking. When $\delta \neq 0$, S^z is no longer conserved, enabling quadrupolar excitations with $\Delta S^z = -2$ to hybridize with dipolar modes. As a consequence, previously "dark" quadrupolar bound states acquire finite dipolar character and become visible in INS measurements.

To illustrate this idea, we adopt the model parameters relevant to $K_2Co(SeO_3)_2$ and compute the corresponding INS intensity using the THED method, both with and without the U(1)-breaking term. Figure S8 compares the two cases: the left panel shows the spectrum for the U(1)-symmetric case ($\delta = 0$), while the right panel includes a finite δ that explicitly breaks the symmetry. As anticipated, breaking U(1) symmetry allows the quadrupolar bound state to acquire dipolar character, leading to finite spectral weight and making it detectable in INS.

Crucially, no corresponding spectral feature is observed at the predicted energy in the experimental data for $K_2Co(SeO_3)_2$, strongly suggesting that the system preserves U(1) symmetry to a very good approximation. This observation supports the conclusion that $K_2Co(SeO_3)_2$ is accurately described by the Hamiltonian proposed in Refs. [3, 4], even though a U(1)-breaking

term is symmetry-allowed. This analysis, made possible by the interpretability and efficiency of the THED method, highlights its power in constraining microscopic Hamiltonians directly from spectroscopic data.

SUPPLEMENTARY MOVIES

Supplementary Movie 1. Bound and resonant two-magnon dynamics in the UUD phase. Video files *dynamical_wave_function_Δ_(5.0,1.0).mp4* show the time evolution of the normalized |transition amplitude|^2 [Eq. (S4)] for $\psi(\boldsymbol{r}_0;\alpha_0,\beta_0)$. For $\Delta = 5$ the two spin flips remain bound (with occasional tunneling), while for $\Delta = 1$ the pair forms a short-lived resonance that decays into two magnons.

[1] A. O. Scheie, Y. Kamiya, H. Zhang, S. Lee, A. J. Woods, M. O. Ajeesh, M. G. Gonzalez, B. Bernu, J. W. Villanova, J. Xing, Q. Huang, Q. Zhang, J. Ma, E. S. Choi, D. M. Pajerowski, H. Zhou, A. S. Sefat, S. Okamoto, T. Berlijn, L. Messio, R. Movshovich, C. D. Batista, and D. A. Tennant, Physical Review B **109**, 014425 (2024).

[2] T. Fukuhara, P. Schauß, M. Endres, S. Hild, M. Cheneau, I. Bloch, and C. Gross, Nature **502**, 76 (2013).

[3] M. Zhu, V. Romerio, N. Steiger, S. D. Nabi, N. Murai, S. Ohira-Kawamura, K. Yu. Povarov, Y. Skourski, R. Sibille, L. Keller, Z. Yan, S. Gvasaliya, and A. Zheludev, Physical Review Letters **133**, 186704 (2024).

[4] M. Zhu, L. M. Chinellato, V. Romerio, N. Murai, S. Ohira-Kawamura, C. Balz, Z. Yan, S. Gvasaliya, Y. Kato, C. D. Batista, and A. Zheludev, npj Quantum Materials **10**, 74 (2025).
